# Facultative apomixis and development of fruit in a deciduous shrub with medicinal and nutritional uses

**DOI:** 10.1093/aobpla/plv098

**Published:** 2015-08-18

**Authors:** Yash Mangla, Manju Chaudhary, Himshikha Gupta, Rakesh Thakur, Shailendra Goel, S. N. Raina, Rajesh Tandon

**Affiliations:** 1 Department of Botany, University of Delhi, Delhi 110 007, India; 2 Amity Institute of Biotechnology, Amity University, Noida, Uttar Pradesh 210 303, India

**Keywords:** Agamospermy, Leh berry, nucellar embryony, reproductive biology, sea buckthorn

## Abstract

Knowledge of reproductive biology of plants is crucial to understand their natural mode of propagation, which may aid in conservation and crop improvement. The reproductive details are also crucial for beginning the cultivation of a potential crop on a commercial scale. Fruits of sea buckthorn, *Hippophae rhamnoides*, are used in a variety of medicinal and nutritional products. So far, fruits are collected from the female plants in the wild. It is known that the species fruits profusely and also propagates by forming root suckers, but the details of sexual reproduction are not available. We investigated the mode of reproduction and development of fruits from natural populations of sea buckthorn. Megasporogenesis and megagametogenesis were studied through resin-embedded sectioning and ovule-clearing methods, and fruit development through histochemistry. The study of mitosis and male meiosis showed that the plants at the site were diploid (2*n* = 2*x* = 24). The embryo sac may develop either through the monosporic pathway and differentiates into ‘Polygonum type’ or aposporously into ‘Panicum type’. The embryo may develop by sexual and adventitious pathways. Thus, sea buckthorn is a facultative apomict. The occurrence of diverse reproductive pathways assures the possibility of generation of novel genotypes through sexuality, while apomictic reproduction maintains adaptive genotypes and ensures reproduction in the absence of pollination. Anatomical details suggest that the fruit of sea buckthorn may be appropriately described as a pseudo-drupe.

## Abstract (precis)

The Himalayan Sea buckthorn, *Hippophae rhamnoides* (Elaeagnaceae) has multifarious uses and employs diverse pathways to reproduce. The species happens to be a facultative apomict, as some seeds are formed without sexual mode (adventive embryony). The combination of sexual and apomictic mode seems to provide reproductive assurance to the plants amidst a less efficient, wind pollination mode. The study provides cytogenetic evidence of the diploid nature of plants and thus adds a new example to the list of known diploid taxa that are apomictic.

## Introduction

Commercial cultivation of potential crop species requires a detailed knowledge of the associated reproductive events. Among the key reproductive features, information on pollination mechanism and breeding system are essential to design a species-specific sexual hybridization strategy in a breeding programme (Acquaah 2007; Shivanna and Tandon 2014). Both the features directly influence the fecundity and are selected in response to the ecological conditions in which the species occurs. In extreme ecological conditions and uncertain pollination environments, the combination of sexual and asexual reproductive modes is likely to be favoured to maximize fitness (Eckert 2002; Allem 2003; Vallejo-Marín *et al.* 2010). The prevalence of multiple reproductive modes in such species is an indication of imparting reproductive assurance. Whereas the vegetative mode generates the clones, the sexual mode engenders heterogeneity and ensures extensive dispersal (Allem 2003; Vallejo-Marín *et al.* 2010; Horandl and Hojsgaard 2012). The facultative apomictic plants, which combine agamospermy with sexuality, exploit the benefits of wider dispersal of clones as well as the segregants through seeds (Peck and Waxman 2000; Richards 2003; Ortiz *et al.* 2013).

The Himalayan sea buckthorn (*Hippophae rhamnoides*), a predominantly dioecious thorny shrub, has gained popularity as a potential crop. It occurs naturally in the alpine zones of temperate and subtropical parts of the World including Europe (Britain, France, Finland, Sweden, etc.), Russia, Central Asia, India and China (Bartish *et al*. 2002; Mabberley 2008). Fruits of sea buckthorn are highly nutritious and their extract has several medicinal properties (Cakir 2004); the fruit juice contains a significant amount of carbohydrates, amino acids, essential fatty acids and vitamins (Zeb 2004; Dhyani *et al.* 2010; Bal *et al.* 2011). The most valuable product of sea buckthorn is oil, extracted from its fruits and seeds.

In many countries of its occurrence including India, there are no commercial plantations of sea buckthorn and so far, the fruits are collected from the wild. In general, owing to a strong faith of people in alternative medicines (Natesh 2001), there has been a considerable rise in the popularity of various products made from its fleshy fruits (Bal *et al.* 2011). Consequently, efforts are underway to bring the species under major cultivation to sustain the supply of raw material, which can be obtained only from the female plants. Analysis of the breeding system and fruit development of the species is a requisite in these attempts and important for the future genetic improvement programme of the species.

In India, Himalayan sea buckthorn or Leh berry is *H. rhamnoides* ssp. *turkestanica*, which exhibits lower intra-population diversity than the other two species, *H. salicifolia* and *H. tibetana*, recorded from the Himalayan region (Raina *et al.* 2012). Sea buckthorn is known to reproduce vegetatively by forming root suckers and sexually by means of seeds (Mangla and Tandon 2014). Profuse clonal reproduction by means of root suckers results in the formation of extensive patches of the plants, and often of the same gender. Our previous study on the pollination mechanism in the species has shown that plants are wind pollinated and pollen flow is effective up to a short distance (Mangla and Tandon 2014). Surprisingly, the isolated female patches of the plant located beyond the effective range of pollen flow also exhibit a considerable amount of fruit set. Detailed investigation on the development of fruits and embryogeny was carried out. Here, we demonstrate that the species is a facultative apomict at the site, as the reproductive mode combines sexual and agamospermous pathways.

## Methods

### Study site

The study was carried out during the peak flowering phase (second and third week of April) of the species for two seasons (2009 and 2011) in two natural populations located at Choglamsar (CV, 34°05.236′N, 077°36.090′E) and Sindhu Darshan (SD, 34°05.269′N, 077°36.687′E) in the Leh–Ladakh region, Jammu and Kashmir, India. Female plants (*n* = 15 in each population) were randomly marked each time in each population during the two seasons to perform the bagging experiments. The voucher specimens for this study (Mangla 14221) were deposited at the Herbarium of the University of Delhi (DUH).

### Ontogeny of the female gametophyte

The development of female gametophyte (course of megasporogenesis and megagametogenesis) was studied from the female flowers collected at (i) 3 and 1 day before anthesis, (ii) on the day of anthesis and (iii) 1 and 3 days after anthesis. The details of anthetic stages of the female flowers have been recorded in our earlier work on the species (Mangla and Tandon 2014). For semi-thin sections, the flowers were collected and fixed in Karnovsky's fixative (Karnovsky 1965), dehydrated in alcohol series and embedded in glycol methacrylate. Sections were cut using a rotary microtome (AO Spencer, USA) and stained with toluidine blue O (Sigma, pH 4.4; Feder and O'Brien 1968). For ovule clearing, the flowers were fixed in 3.7% formaldehyde, 5% acetic acid, 50% ethanol (FAA) (Ruzin 1999). Ovules were dissected out from the pistils under a stereomicroscope, treated with lactic acid solution (saturated with chloral hydrate) for 24 h. The ovules were then washed three times in 70 % ethanol (5 min each), transferred to the clearing solution (Herr 1971) and kept for 7 days at 28 °C. The cleared ovules were observed under a differential interference contrast microscope (Carl Zeiss, Germany).

### Fruit set

The extent of fruit set through open pollination in an infructescence was determined by counting the fruits from the marked inflorescences (*n* = 30 each in CV and SD, *n* = 2 from each plant). The fruit to flower ratio was computed by dividing the average number of fruits formed in an infructescence (*n* = 30 each in CV and SD, *n* = 2 from each plant) with the average number of flowers borne in an inflorescence.

To ascertain the possibility of apomixis, female inflorescences (*n* = 324 at CV and 417 at SD) were bagged without pollination, 3 days before the anthesis of flowers. Due to the small size of the female flowers, the entire inflorescence was bagged. Some of the bagged inflorescences (*n* = 42 at CV and 95 at SD) were fixed in FAA or Karnovsky's fixative at intervals of 1, 2, 3, 5 and 6 days after bagging (DAB) and processed for sectioning or ovule clearing as specified above. The remaining tagged and bagged inflorescences were left for fruit formation.

The difference in the outcome (fruit set, dependent variable) of the two treatments (open pollination vs. bagging experiment) and their effect in two populations and two seasons (fixed factors) was analysed through ANOVA. Fruit set data from each plant were averaged for each type of treatment (open-pollinated or bagging) and the plants were considered as cases. The percentile fruit set data were normalized by square-root arcsine transformation before performing the analysis. Statistical analysis was carried out using the SPSS16 package (SPSS, Inc. 2007).

### Estimation of ploidy

To determine the ploidy level of the plants at the site, meiosis and mitotic preparations were analysed. For this, the male flower buds and seed materials were collected. Fresh root tips were harvested from the young plants grown from seeds. The root tips were washed and pre-treated with cold water at 4 °C for 24 h. The pre-treated root tips were fixed in freshly prepared acetic alcohol (1 : 3) for 24 h. The root tips were washed in distilled water, hydrolysed using 5 N HCl for 1 h at room temperature and stained with 0.5 % Feulgen solution for 1 h. The stained root tips were squashed in 45 % acetic acid and observed under the microscope (Axiscope, Zeiss, Germany). For male meiosis, anthers were squashed in 1 % aceto-carmine and observed under the microscope. To validate the results, 5–10 well-spread chromosome preparations from each population were analysed.

### Ontogeny of fruit and seed

Embryogeny and differentiation of fruit was studied from resin-embedded sections of fruits obtained from open pollination (*n* = 90 fruits, 18 infructescence) and bagging (*n* = 70 fruits, 33 infructescence). The fruits were fixed at various developmental stages (10, 50–70, 70–90 and 100–120 DAB). Sections were stained with 1-anilinonaphthalene-8-sulfonic acid (0.001 %) for localizing the proteins (Mattsson *et al.* 1974) and PAS (periodic acid Schiff's reagent) for polysaccharides and lipids (McGuckin and McKenzie 1958). The measurements of developing fruits were made using a digital Vernier calliper.

Finally, the number of mature fruits formed in the bagged inflorescences (*n* = 268 at CV and 303 at SD) was recorded. Some of the seeds (*n* = 50) obtained from each of the treatments were also germinated to find out the incidence of polyembryony.

## Results

### Natural fruit set

The two populations did not vary in the amount of mean natural fruit set. On an average, 4.44 ± 1.50 fruits were formed in an infructescence. The fruit to flower ratio in female plants was 0.68 (∼68 %), as the average flower production in an inflorescence was 6.14 ± 1.81. The seeds obtained from both the open pollination and bagging showed 100 % germination under laboratory conditions. No incidence of polyembryony was found in the seeds of either type.

### Apomixis

Although significantly lower in amount (*F*_(1,82)_ = 164.87, *P*= 0.001) than the open-pollinated ones (68 %), bagging of unpollinated female flowers in both the populations resulted in fruit set (16 %). The ability of the flowers to develop seeds without pollination indicated the incidence of apomixis. The three-way interaction between seasons, treatment and the populations had no significant effect on the fruit set (*F*_(1,82)_ = 2.155, *P*= 0.146).

### Ploidy of the plants

**Figure 1.**
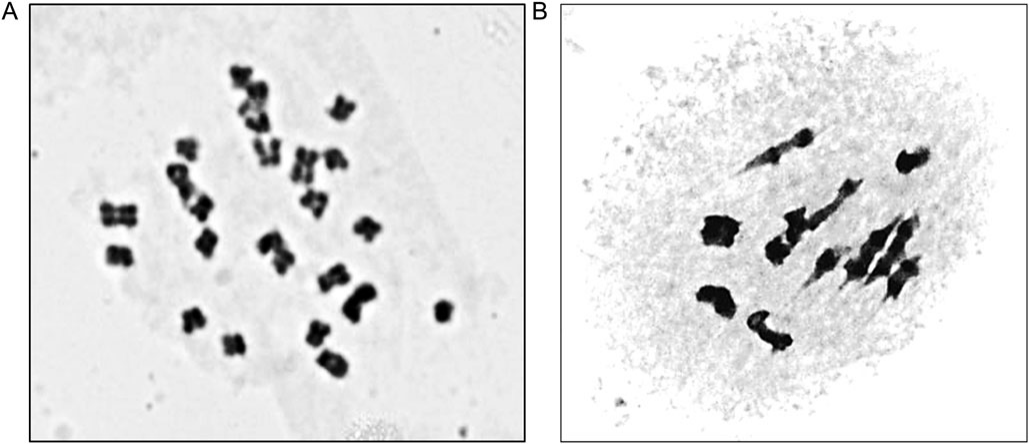
Ploidy of Himalayan sea buckthorn. (A) A representative chromosome preparation from the root tip showing 24 chromosomes. (B) Male meiosis at metaphase I showing 12 bivalents.

The chromosome number in the somatic cells was 2*n* = 24 (Fig. 1A). In male plants, meiosis was normal and a majority (98.7 %) of pollen mother cells showed normal 12 bivalents at metaphase I (Fig. 1B); the remaining cells had 11II + 2I. These cytogenetic details indicate diploid constitution of the species.

### Female gametophyte

**Figure 2.**
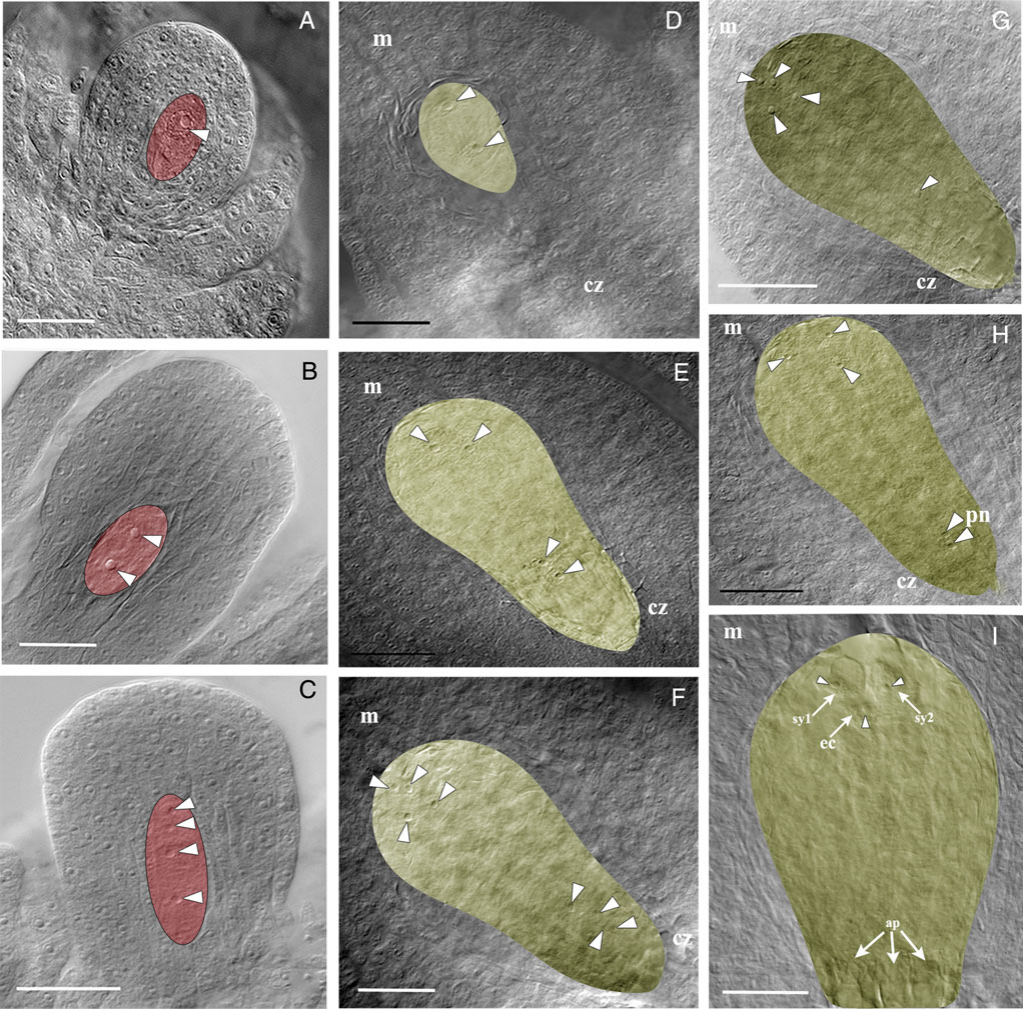
*Polygonum* type of female gametophyte development, as ascertained by ovule clearing. The nuclei are marked with white arrowheads. (A–C) Stages of megasporogenesis. A crassinucellate ovule with MMC (A), at dyad stage (encircled) after meiosis I (B) and a linear tetrad of megaspores after meiosis II (C). The largest megaspore located at the chalazal end remains functional. (D–I) Stages of megagametogenesis. Embryo sacs at two-nucleate (D), four-nucleate (E) and eight-nucleate (F) stage. (G) Embryo sac with five of the eight free nuclei; four nuclei are located towards the micropylar end and one at the chalazal end; the other three nuclei that eventually participate in the formation of three antipodal cells are not in the view. (H) One of the four nuclei from the micropylar end migrates towards the chalazal end; the two free nuclei function as polar nuclei of the central cell. (I) Embryo sac with an egg apparatus (two synergids, sy1 and sy2; and one egg cell) at the micropylar end and three antipodals; the two polar nuclei are not visible in this plane. ap, antipodal; cz, chalazal end; ec, egg cell; m, micropylar end; pn, polar nuclei. Scale bars: (A–C) 100 μm; (D–G) 50 μm; (H and I) 20 μm.

**Figure 3.**
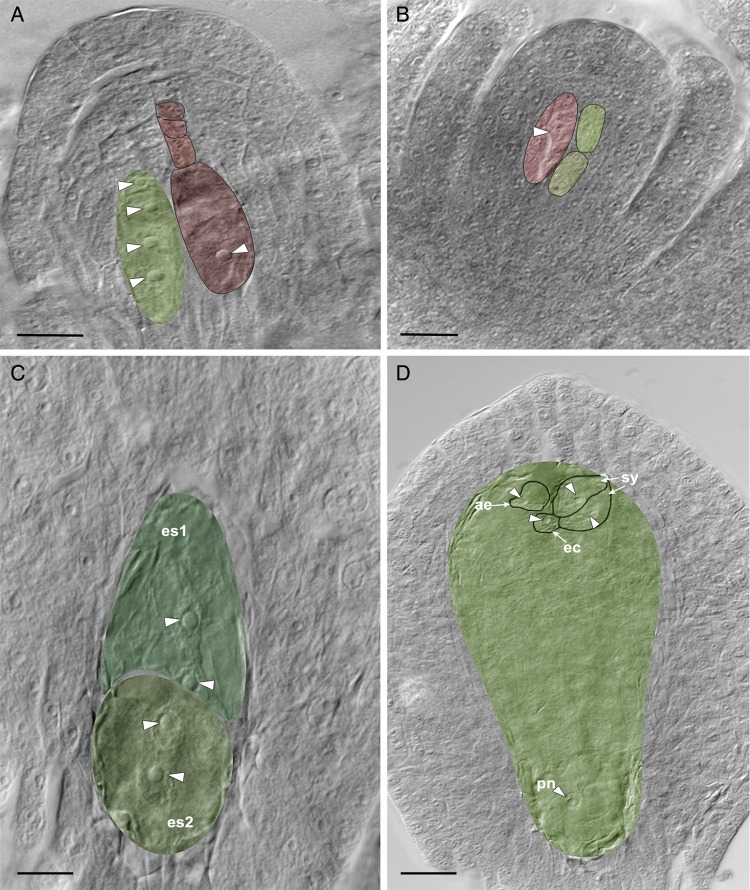
Aposporous mode of embryo sac development; nuclei are indicated by white arrowheads. (A) The presence of linear tetrad of sexual megaspores (red) with a large functional megaspore and four-nucleate aposporous initial (green) in the same ovule. (B) An ovule with a degenerated sexual MMC (arrow) and two developing aposporous initials (arrowheads). (Compare with **Supporting Information—Fig. S1C**.) (C) An ovule with two aposporous embryo sacs (es1 and es2), at a two-nucleate stage. (D) A four-nucleate aposporous embryo sac: two synergids, egg cell and one pn. Note the development of an adventitious embryo (arrow, ae) from the nucellus next to the egg apparatus. sy, synergids; ec, egg cell; pn, polar nucleus. Scale bars: (A and C) 25 μm; (B and D) 20 μm.

The megagametophyte development in sea buckthorn followed the sexual (Fig. 2) as well as the agamospermous routes (Fig. 3) **[see Supporting Information]**. Additionally, young fruits from the bagging treatment exhibited adventive (nucellar) embryogeny (Fig. 5). Based on anatomical observations, the incidence of apomixis (agamospermy) was recorded in 30.22 ± 6.03 % of the ovules (Table 1).

**Table 1.** Quantitative details of various developmental stages of the female gametophyte through sexual and apomictic modes in *H. rhamnoides*. Details mentioned are based on both sectioning and ovule-clearing methods. ND, not distinguishable.

DAB	Stage of female gametophyte			
	Sexual mode		Apomictic mode	
	Stage	Ovules showing sexual mode (%)	Stage	Ovules showing apospory (%)
1	MMC/dyad	ND	MMCs	ND
2	Megaspore tetrad formation/two- to four-nucleate stage of megagametogenesis/four- or eight-nucleate stage of the megagametophyte	78.72	Degeneration of sexual MMC/aposporous initials	21.27
3	Eight-nucleate stage of the megagametophyte/initiation of embryo sac organization	66.66	Two megaspore tetrad/growth of multiple aposporous initials	33.33
5	A mature embryo sac	68	Multiple embryo sacs	32
6	A mature embryo sac	65.72	Multiple embryo sac/multiple egg apparatus	34.28

#### Sexual type

The ovule is crassinucellate type, as the megaspore mother cell (MMC) differentiates deep inside the nucellus (Fig. 2A). Meiotic division in the MMC results in a linear tetrad (Fig. 2B and C). The megaspore cell at the chalazal end remains functional and the other three in a tetrad degenerate. The functional megaspore cell undergoes the first mitotic division; the orientation of the division is longitudinal (Fig. 2D). Further development follows the ‘*Polygonum* pathway’ (Maheshwari 1950) (Fig. 2E–I). Thus, the development of the sexual embryo sac in *H. rhamnoides* is monosporic, resulting in a seven-celled, eight-nucleate embryo sac (Fig. 2H and I).

#### Agamospermous type

The MMC differentiates, but does not enter meiosis. Instead, some of the nucellar cells, the aposporous initials (Fig. 3A and B) **[see Supporting Information]** enlarge and directly function as megaspores without undergoing meiosis. The sexual MMC, which also differentiates simultaneously with the aposporous initial, is usually degenerated (Fig. 3A) **[see Supporting Information]**. Often, the sexual MMC may differentiate up to the tetrad stage. However, with further growth of the aposporous initial(s) (Fig. 3A), the sexual type degenerates **[see Supporting Information]**. Simultaneous divisions in several aposporous initials may result in the formation of multiple embryo sacs in an ovule (Fig. 3C) **[see Supporting Information]**. The mature embryo sac is unreduced, four-celled and four-nucleate (Fig. 3D) **[see Supporting Information]**, and conforms to ‘*Panicum* type’.

#### Embryo and endosperm development

**Figure 4.**
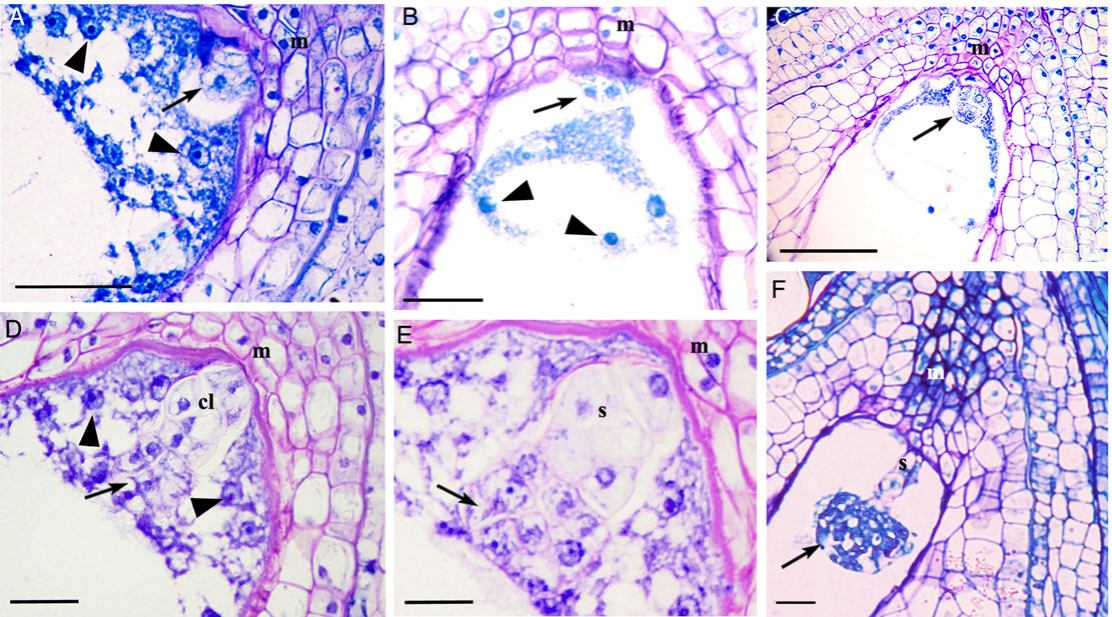
Embryo development through a sexual mode. (A) Longitudinal section of a portion of an ovule with a zygote (arrow) at the micropylar end and a free-nuclear endosperm (arrowheads). (B) Longitudinal section of a portion of the ovule at the two-celled stage of the proembryo (arrow) and free-nuclear endosperm (arrowheads). A four-celled (C, arrow) and eight-celled (D, arrow) proembryo. In the latter, the basal tier is larger than the upper tier of cells (arrow). (E) A 16-celled proembryo with suspensor; the latter being formed from the basal tier of the cells. (F) A globular embryo with a well-developed suspensor. Note that the endosperm is consumed by this stage. cl, basal tier; m, micropylar end; s, suspensor. Scale bars: (A and B) 50 μm; (C–F) 100 μm.

In the sexual type, the zygote does not divide immediately after its formation but undergoes a period of dormancy for ∼6–7 weeks. Meanwhile, the primary endosperm nucleus divides and forms a free-nuclear endosperm (Fig. 4A). The zygote divides longitudinally and forms a two-celled proembryo (Fig. 4B). Thus, the embryogeny corresponds to ‘Piperad type’ (Johri *et al*. 1992). The second division in both the cells is transverse, and forms a four-celled proembryo (Fig. 4C). Subsequently, transverse and longitudinal divisions give rise to an elongated eight-celled proembryo (Fig. 4D). The smaller cells in the upper tier of the octant divide actively to form a 16-celled proembryo (Fig. 4E). Further divisions in the periclinal and anticlinal planes result in a globular embryo and the lower tier of cells differentiate into a suspensor (Fig. 4F). The endosperm is consumed by the time a globular embryo is differentiated (Fig. 4F). The seeds of *H. rhamnoides* are thus ex-albuminous.

#### Nucellar (adventive) embryony

**Figure 5.**
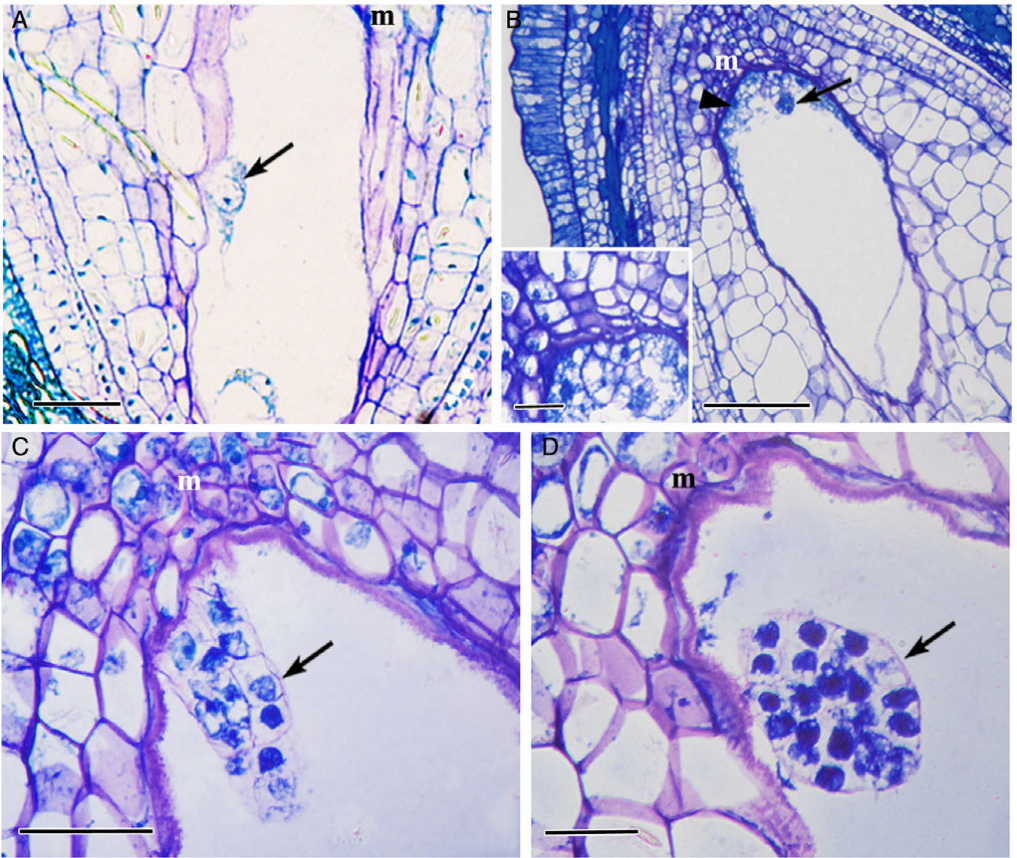
Young fruits with adventitious embryony. (A) A two-celled proembryo (arrow) originating from the nucellus. (B) Early embryo at the four-celled stage (arrow) with free-nuclear endosperm (arrowhead). *Inset*: a magnified view of the proembryo. (C) An elongated multicellular proembryo (arrow). (D) A globular embryo without the suspensor (arrow). m, micropylar end. Scale bars: (A) 500 μm; (B–D) 100 μm.

In the absence of pollination (bagging experiment), the embryo did not develop from the egg cell of the sexual and aposporous megagametophytes. In these cases, the embryo differentiates from the nucellar cells (Fig. 5A). The embryo originates at positions other than at the micropylar end. In aposprous ovules, the embryo sac that does not contribute to embryo formation invariably degenerates. The proembryonal cells undergo divisions and form a four- to eight-celled proembryo (Fig. 5B and C). Further divisions in the proembryo resulted in the formation of globular-shaped ‘adventitious embryos’, which lacked a suspensor (Fig. 5D). The endosperm remains free-nuclear and persists only up to the four-celled stage of the adventive embryo (Fig. 5B). The endosperm development in these fruits was likely an autonomous one because pollination was prevented through bagging.

### Structure and development of fruit

**Figure 6.**
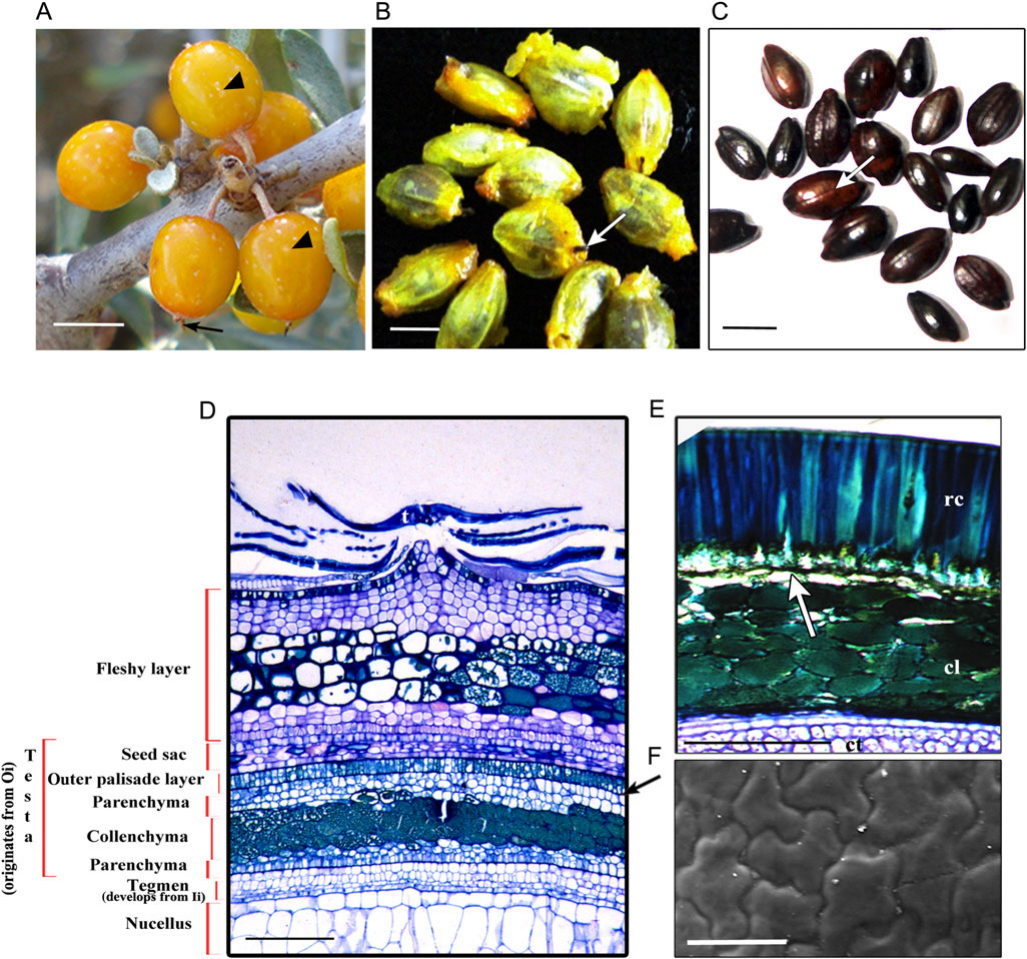
Fruit and seed development. (A) An infructescence with mature fruits 120 days after pollination (DAP). Arrow indicates the dried persistent stigma (seed tail) and arrowheads the peltate trichomes on the fleshy perianth. (B) Mature seeds encased within the yellowish membranous carpellar wall (seed sac). Arrow indicates the seed tail. (C) Mature seeds without the seed sac. The shallow groove in the seeds is noticeable (arrow). (D) A part of the longitudinally cut section of a young fruit (70 DAP) with wall layers of the fruit and the seed. Note the palisade-like layer (arrow) and differentiation of parenchyma–collenchyma–parenchyma layers in the Oi (testa). The collenchyma accumulates phenolics. The Ii of the ovule differentiates into a tegmen. (E) A part of the longitudinal section of a mature seed coat. The testa is well developed and is composed of an outer palisade-like layer with thickenings and collenchyma with phenolics; crushed parenchyma (arrow) is sandwiched between the two layers. The tegmen is relatively inconspicuous and is represented by one or two layers of parenchymatous tissue. (F) A part of the scanning electron micrograph depicting the sporoderm pattern of a mature seed. cl, collenchyma; ct, cotyledon; ec, elongated cells of testa; Ii, inner integument; Oi, outer integument; t, trichomes. Scale bars: (A) 4 mm; (B and C) 2 mm; (D) 500 μm; (E) 100 μm; (F) 25 μm.

Fruit set becomes apparent 10 days after pollination. Afterwards, no increase in size of the female flowers occurs till 50 days after fertilization (DAF). The ripe fruits (110–120 DAF) are yellow or orange-red (Fig. 6A), single-seeded and measure 8.05 ± 0.43 mm in length. The seed are ovate, brownish-black with a shallow ventral groove (Fig. 6C) and measure 4.509 ± 0.38 mm in length; 100 seeds weighed 113.7 mg.

At 10 DAF, the perianth tube becomes a part of developing fruit and begins to accumulate phenolics, oils, polysaccharides and proteins. After 60–70 DAF, the perianth tube became 8- to 10-cell-layers thick and vacuolated, and developed into a fleshy green layer. In mature fruits, the fleshy perianth had 12- to 15-cell layers and a few cells accumulated phenolics. The epidermis of the perianth tube bears numerous peltate trichomes that are gradually shed during the development of fruit and, eventually, only a few of them persist over the mature fruits (Fig. 6A). At the time of pollination, the carpel wall is six- to seven-cell-layers thick at the ovarian region. However, it gradually shrinks and forms a thin papery covering, known as pericarp, over the mature seed (Fig. 6B and D). It remains firmly attached to the mature seed towards the inner side but remains separated from the peripheral fleshy layer with an intervening air space. The persistent dried stigma remained attached to this layer forms a tail-like structure, known as ‘seed tail’ (Fig. 6B).

Both the inner integument (Ii) and the outer integument (Oi) contribute to the formation of a seed coat. The Oi differentiates into testa and Ii develops into tegmen. During the initial phase of fruit development (50–70 DAF), the epidermal cells of the Oi elongate radially and develop thickenings (Fig. 6D); the radial cells are more elongated towards the micropylar end. At 100 DAF, the Oi also differentiates into testa. Here, the three- or four-cell layer thick collenchyma is sandwiched between the parenchyma cells (four- or five-cell layers) on each side (Fig. 6D).

At maturity, the seed coat becomes brownish-black (Fig. 6C) and is covered with a yellowish-orange fleshy layer. The mature seed carries a typical dicotyledonous embryo. The seed coat differentiates into three recognizable layers. The outer layer is derived from the epidermis of the Oi and is composed of elongated cells filled with tannins and radial-wall thickenings (Fig. 6E). Thus, the seeds of sea buckthorn are exotestal (derived from Oi). In the surface view, these cells appear irregular in shape (Fig. 6F). The second layer is seven- to eight-cells thick and collenchymatous (Fig. 6E). Divisions in the collenchymatous cell layers and growth in the outermost thick-walled cells crush the parenchyma of the Oi. Together, these layers form a ‘testa’ (Fig. 6E). The Ii does not undergo further differentiation and directly forms the unspecialized tegmen (the third layer) of the seed. Periclinal divisions result in increment in the size of the tegmen, which covers the developing seed. In a mature seed, it is five- to seven-cell-layers thick over the radicle and thinner (three- or four-cell-layers thick) around the cotyledons (Fig. 6E).

## Discussion

### Facultative apomixis

Fruit set in anemophilous plant species is generally low due to the limitation of either pollen or pollination (Niklas 1985; Paw and Hotton 1989; Ackerman 2000; Friedman and Barrett 2009; Mangla and Tandon 2011). Contrastingly, wind-pollinated *H. rhamnoides* at the study sites exhibited considerably greater fruit set ∼68 %. Moreover, the observed value of fruit set was greater than the expected fruit set, as only ∼60 % of the stigmas were found to be open-pollinated in an earlier investigation at the same sites (Mangla and Tandon 2014). This difference in the values could be attributed to the possibility of an apomictic mode of seed development.

The apomictic mode of reproduction disperses clonality through seeds and assures reproduction of the species. In many species, the apomictic mode may coexist with sexual and thus permit heterogeneity in combination with reproductive assurance. Bagging treatment in female plants clearly demonstrated that fruit set may be achieved without pollination also (adventive embryony), although to a lower extent than that from open pollination. The embryological evidence showed that embryos may develop through (i) syngamy (∼54 %), (ii) an aposporous mode (∼30 %) and (iii) nucellar (adventitious) embryony (sporophytic apomixis, ∼16 %). Thus, the reason for greater seed set in the species at the sites could be due to an apomictic mode in addition to the sexual mode. These findings suggest that plants of *H. rhamnoides* at the site are facultative apomictic. Moreover, the formation of twin embryo sacs in an ovule is usually associated with the prevalence of apomixis (Kaur *et al.* 1978).

Adventive embryony is the most common route to apomixis in angiosperms, which often coexists with the sexual mode of reproduction. There are several plant species where apospory co-occurs with adventive embryony such as *Malus*, *Pyrus*, *Allium* and *Ochna* (Naumova 2008). Apomixis is generally present in association with polyploidy. Polyploidy is considered as the maintenance and stabilization force of apomixis (Bicknell and Koltunow 2004; Naumova 2008), although there are also reports of diploid apomicts such as in *Boechera* (Aliyu *et al.* 2010; Voigt *et al.* 2012), *Brachiaria decumbens* (Naumova *et al.* 1999) and *Paspalum rufum* (Siena *et al.* 2008). This occurrence of diploid apomicts indicates that polyploidy is not an absolute requirement for apomixis, even though it may enhance the prevalence of apomixis (Bicknell and Koltunow 2004). The present study highlights the combination of sexuality and asexuality (apospory and adventive embryony) in diploid sea buckthorn. Lower fruit set through agamospermy (∼16 %) when compared with sexual reproduction (∼68 %) in the species is in agreement with other diploid and facultative apomictic taxa like *B. decumbens* (10–15 %) (Naumova *et al.* 1999) and *P. rufum* (8.8–28.8 %). In the latter species, ovules may possess both a sexual and an ‘aposporous-like’ embryo sac (Siena *et al.* 2008). Thus, *H. rhamnoides* is an addition among the limited number of known diploid facultative apomicts.

Environment and genetic factors are the two important factors that may lead to loss of sexual reproduction in clonal populations (Eckert 2002; Vallejo-Marín *et al.* 2010). In diploid species, sexuality may avoid the accumulation of deleterious mutations over generations (Muller ratchet) (Richards 2003). However, sometimes avoidance of sexual reproduction is to prevent the break-up of the favourable gene combinations attained by earlier selection (Ortiz *et al.* 2013), as sexual reproduction cannot always bring favourable alleles together via recombination. This results into the selection of mechanism to avoid sexual reproduction and has an advantage of clonal multiplication, covering vast areas with single and highly adapted genotypes (Johri 1984; Richards 2003; Wesche *et al.* 2005; Ortiz *et al.* 2013). These assertions favour multiple modes of clonal reproduction to coexist along with sexuality, as in diploid *H. rhamnoides*, which is spread over a vast area in the region. Moreover, asexuality minimizes the cost of reproduction, thereby resulting in higher quantities of offspring (Smith 1978; Bell 1982; Horandl and Hojsgaard 2012) in inclement environmental conditions.

### Fruit and seed development

The morphological and anatomical details showed that the fruit of sea buckthorn is fleshy, anthocarpic, non-arillate, single-seeded and indehiscent. The seed along with the seed sac (dried pericarp) is attached from a single point, as there is a conspicuous air space between the fleshy perianth and the remaining seed. The study suggests that the type of fruit in sea buckthorn is not a true berry and its description does not completely fit into any of the botanical classification of fruits (Radford 1974; Black *et al.* 2006). In sea buckthorn, the perianth tube alone contributes to the formation of the fleshy layer. The ovary wall (carpellar wall), instead, contributes to a thin papery pericarp known as the seed sac. In this context as well, its fruits cannot be termed ‘berry’. The other two main descriptions of the fruit, i.e. a drupe or a nut, are also not possible, because a well-differentiated pericarp, with noticeable exocarp, mesocarp and endocarp and stony endocarp, are lacking in this species.

A detailed investigation made on *H. rhamnoides* cv. Indian summer (Harrison and Beveridge 2002) suggested that fruits of sea buckthorn should be described as achene with a woody seed coat, enclosed in a fleshy hypanthium. In view of a clear disagreement with the other possible types of fruits discussed above, it is tempting to use the term ‘achene’ for sea buckthorn to some extent because the presence of one seed in a fruit, indehiscent nature, attachment of seed from a single point and development from a unilocular ovary are in accordance with an ‘achene’. However, certain features may counter this premise. An achene by definition does not have a well-differentiated seed coat (Black *et al.* 2006), while in sea buckthorn, the seed coat possesses a distinguishable testa and tegmen. Additionally, the fleshy region does not develop from a hypanthium that essentially involves basal fusion of sepals, petals and stamens. The two perianth lobes in female flowers of sea buckthorn are rather connate from its sub-apical position and form a tube; the latter is free from the gynoecium. In the absence of stamens or its rudimentary structures in female flowers (Mangla *et al.* 2013), it is not clear if the hypanthium is present in the flowers of pistillate plants of sea buckthorn. Even though, it is not the basal region alone but the entire perianth tube that contributes to the fleshy region of the fruit. Moreover, the other typical feature of an achene, that is the dry nature of the fruit, contrasts with the fleshy fruits in sea buckthorn.

In related genera such as *Shepherdia* and *Elaeagnus*, fruits are termed ‘acrosarcum’ (seed embedded in fleshy pulp without a distinct endocarp) and ‘pseudo-drupe’ (the anthocarp differentiates from the pericarp and lacks an endocarp), respectively (Spjut 1994). In sea buckthorn, the carpellar fruit is indehiscent, but the dispersal unit (diaspore) is represented by a fleshy and showy perianth around the carpel wall. The fruits of sea buckthorn resemble *Elaeagnus* and thus may be preferably described as pseudo-drupe because, as in false fruits, there is only one seed and the other floral organs also contribute to the formation of a diaspore.

## Conclusions

*Hippophae rhamnoides* is a predominantly dioecious taxon, which reproduces under harsh ecological conditions. Combination of sexual and agamospermous reproductive pathways in the species appears to buffer the possible limitations of pollen and mates in an anemophilous and dioecious plant species by producing diverse segregants under extreme environments. The occurrence of multiple reproductive modes in the species appears to ensure reproductive assurance and would be of commercial advantage to sustain the yield. In order to establish any deviation in the reproductive strategy of the species, it would be important to extend the study to the other regions of its natural distribution range.

## Sources of Funding

The present work was financially supported by the University Grants Commission, India (F. No. 37-405/2009 SR) and R&D grant from the University of Delhi.

## Contributions by the Authors

Y.M. and R.T. have made equal contributions in conducting the research. M.C., H.G. and R.Th. carried out the cytogenetic work. Y.M., S.G., S.N.R. and R.T. were involved in planning the research and writing the manuscript. All authors read and approved the manuscript.

## Conflict of Interest Statement

None declared.

## Supporting Information

The following additional information is available in the online version of this article –

**Figure S1.** Various stages of aposporous embryo sac development.

Additional Information
