# Supplementary material for: Facultative apomixis and development of fruit in a deciduous shrub with medicinal and nutritional uses
Source: AoB Plants. 2015 Aug 18;7:plv098. doi: 10.1093/aobpla/plv098 (PMC4589571; doi:10.1093/aobpla/plv098)
Supplement: Additional Information [file supp_7_plv098_index.html]

Facultative apomixis and development of fruit in a deciduous shrub with medicinal and nutritional uses — Additional Information 

# Facultative apomixis and development of fruit in a deciduous shrub with medicinal and nutritional uses

## Additional Information

Additional Information

- Additional Information - Docx file
