# Supplementary figures and images for: Facultative apomixis and development of fruit in a deciduous shrub with medicinal and nutritional uses
Source: AoB Plants. 2015 Aug 18;7:plv098. doi: 10.1093/aobpla/plv098 (PMC4589571; doi:10.1093/aobpla/plv098)

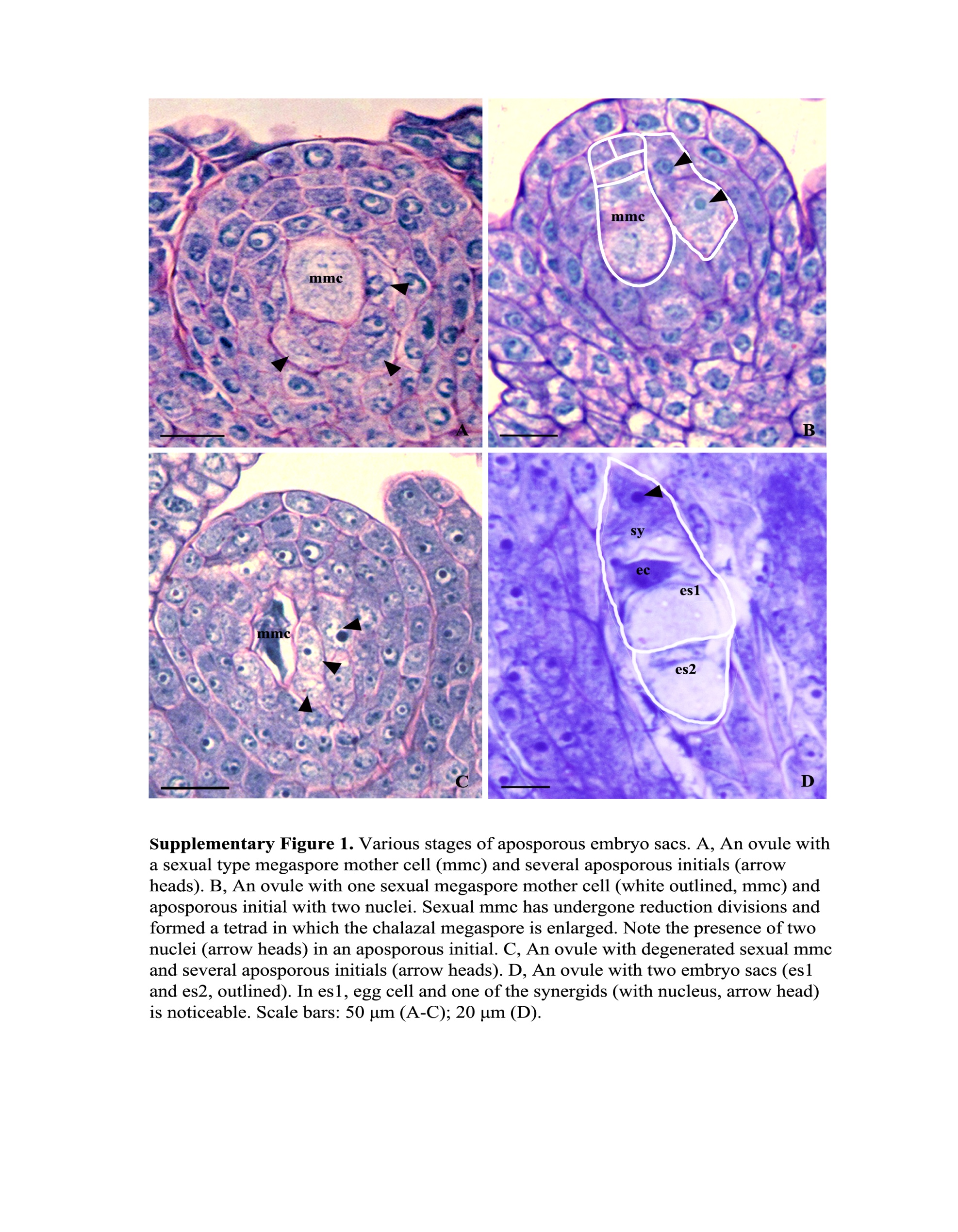

Supplement: Additional Information [file supp_plv098_plv098supp.docx]
